# Multiplexed long-read plasmid validation and analysis using OnRamp

**DOI:** 10.1101/gr.277369.122

**Published:** 2023-05

**Authors:** Camille Mumm, Melissa L. Drexel, Torrin L. McDonald, Adam G. Diehl, Jessica A. Switzenberg, Alan P. Boyle

**Affiliations:** 1 Department of Human Genetics, University of Michigan, Ann Arbor, Michigan 48109, USA;; 2 Department of Computational Medicine and Bioinformatics, University of Michigan, Ann Arbor, Michigan 48109, USA

## Abstract

Recombinant plasmid vectors are versatile tools that have facilitated discoveries in molecular biology, genetics, proteomics, and many other fields. As the enzymatic and bacterial processes used to create recombinant DNA can introduce errors, sequence validation is an essential step in plasmid assembly. Sanger sequencing is the current standard for plasmid validation; however, this method is limited by an inability to sequence through complex secondary structure and lacks scalability when applied to full-plasmid sequencing of multiple plasmids owing to read-length limits. Although high-throughput sequencing does provide full-plasmid sequencing at scale, it is impractical and costly when used outside of library-scale validation. Here, we present Oxford nanopore-based rapid analysis of multiplexed plasmids (OnRamp), an alternative method for routine plasmid validation that combines the advantages of high-throughput sequencing's full-plasmid coverage and scalability with Sanger's affordability and accessibility by leveraging nanopore's long-read sequencing technology. We include customized wet-laboratory protocols for plasmid preparation along with a pipeline designed for analysis of read data obtained using these protocols. This analysis pipeline is deployed on the OnRamp web app, which generates alignments between actual and predicted plasmid sequences, quality scores, and read-level views. OnRamp is designed to be broadly accessible regardless of programming experience to facilitate more widespread adoption of long-read sequencing for routine plasmid validation. Here we describe the OnRamp protocols and pipeline and show our ability to obtain full sequences from pooled plasmids while detecting sequence variation even in regions of high secondary structure at less than half the cost of equivalent Sanger sequencing.

Cloning of recombinant DNA into plasmid vectors is a fundamental tool of molecular biology that has been central to many discoveries in genetics for decades, including the first sequencing of the human genome (International Human Genome Sequencing Consortium 2001). It continues to underpin modern-day research in genomics, protein expression and purification (Rosano and Ceccarelli 2014), transcriptional regulation (Inoue and Ahituv 2015), and gene therapies (Mali 2013). However the standard for plasmid sequence validation, an important step in cloning owing to the error-prone nature of recombinant assembly (Conley et al. 1986; Potapov and Ong 2017), is still Sanger sequencing, a PCR-based method invented in 1977 (Sanger et al. 1977).

Sanger sequencing uses a PCR-amplification-based approach to obtain base-pair resolution of DNA sequence in stretches of up to 900 bp (Sanger et al. 1977). Despite being an important tool for simple, low-throughput sequence validations, Sanger also has a number of limitations. These include the need to synthesize target-specific primers, inaccuracy in long mononucleotide stretches (Shinde et al. 2003), difficulty sequencing through regions with strong secondary structure (such as repetitive elements), and a limit of ∼900-bp sequence output per run (Stranneheim and Lundeberg 2012). Although the 900-bp limit can be addressed by tiling multiple sequencing runs across the same plasmid, this requires synthesis of multiple primers and quickly becomes expensive and laborious when applied to multiple transformants. As a result, typical Sanger validation protocols involve sequencing only the portion of a plasmid that was most recently modified or that contains protein-coding sequence, and routine validation of full plasmid sequences after cloning is not standard practice. However, we are increasingly recognizing the potential impacts that plasmid backbone sequences, structural elements, and bacterial sequences, which are not commonly sequence-validated and can vary widely between plasmids, have on regulation and activity of other plasmid components. Most plasmids contain multiple elements that contribute to function (Williams et al. 2009; Kittleson et al. 2012), including bacterial sequences (Muerdter et al. 2018), which may accumulate undetected errors as a result of spot-check sequencing approaches and can impact downstream function.

High-throughput short-read sequencing (HTS) does allow for simultaneous sequencing of large numbers of plasmids and provides full plasmid sequences (Gallegos et al. 2020). However, HTS is cost-prohibitive outside of large-scale approaches, and sample pooling coordination, indexing compatibility issues, equipment cost, and turnaround time are major barriers to its widespread adoption and make it unsuited for routine plasmid validation. Additionally, HTS does not allow for detection of variation outside of unique regions of plasmids in libraries owing to the inability to uniquely map short reads to an individual plasmid (Liu et al. 2012).

With the advent of Oxford Nanopore Technologies’ (ONT) benchtop long-read sequencing platform, a new option has become available for plasmid validation. Nanopore sequencing platforms can generate reads on the order of megabases and have been used in a variety of applications, including the resolution of previously intractable complex structural variation, whole-genome sequencing, targeted enrichment sequencing, clinical diagnostics, RNA sequencing, and metagenomics (Sanchis-Juan et al. 2018; Bowden et al. 2019; Gilpatrick et al. 2020; McDonald et al. 2021; Wang et al. 2021). Nanopore's read length allows for validation of entire plasmid sequences in a multiplexed format, unlike Sanger, and the low cost of the platform relative to HTS makes it more accessible. Although some groups have applied nanopore to the task of plasmid sequencing, they use transposase- and barcode-based de novo assembly approaches (Currin et al. 2019; Emiliani et al. 2022; Brown et al. 2023). Importantly, the approaches used in these studies all require bioinformatic expertise in order to properly analyze data and interpret results from libraries prepared using these methods. To take steps toward more widespread adoption of full-plasmid sequencing using nanopore, accessibility is crucial to address. Researchers who do routine plasmid validation are likely to be bench scientists. Therefore, in order to improve accessibility and utility to a broader population, it is important to have protocols and analysis tools that not only allow for rapid, easy analysis of nanopore plasmid data but also provide analysis outputs that make interpretation as easy as it is for the current validation standard, Sanger sequencing.

Here we present Oxford nanopore-based rapid analysis of multiplexed plasmids (OnRamp), a tool that leverages ONT's long-read technology to obtain full sequences of pooled plasmids. OnRamp addresses the need for an approach that is simpler and more cost-effective than HTS, while providing full plasmid sequences at medium-throughput scale in a rapid and amplification- and barcode-free manner at under $1.25 per kilobase, less than half the cost of equivalent Sanger sequencing. OnRamp comprises both custom protocols and an analysis pipeline for ONT long-read pooled plasmid data, available through the OnRamp web app (https://onramp.boylelab.org/). OnRamp uses a reference-based approach that allows for the viewing of reference-consensus alignments, alignment quality scoring for rapid identification of correct versus incorrect plasmids, and a view of individual read alignments for interpretation of base call confidence and detection of subpopulation-level variation, all through the OnRamp web application, making interpretation of sequencing results accessible and simple. Here, we describe custom plasmid preparation protocols for use with OnRamp, describe testing of the OnRamp pipeline using simulated read data, and show detection of variation using real plasmid data across plasmid pools containing both dissimilar and highly similar (clonal) plasmid sequences.

## Results

### OnRamp protocols and pipeline

**Figure 1.**
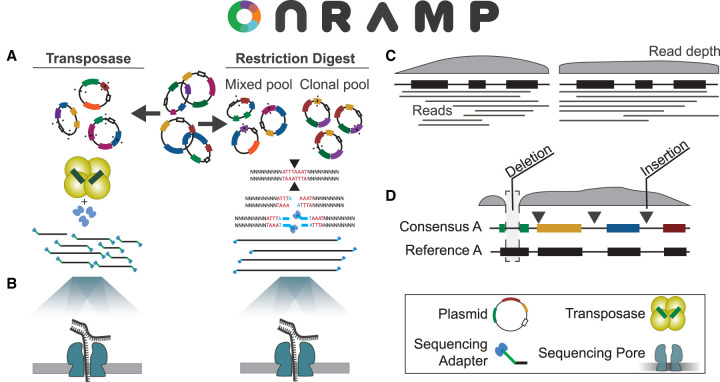
OnRamp protocol and pipeline. Pooled plasmids have nanopore adapters added by transposase or by digestion and ligation (*A*) and then are sequenced (*B*). (*C*) Base-called reads are provided to the OnRamp web app, which generates consensus sequences. (*D*) Consensus sequences are then aligned to user-provided references to identify variation.

The OnRamp protocols use ONT's nanopore sequencing platform, which requires ligation of DNA ends with specialized adapters used to facilitate sequencing. Our method is unique in that it leverages full-length plasmid reads for assembly and does not require barcodes for multiplexed runs, which allows for rapid and simple sample preparations. Here we provide two methods for plasmid pool preparation for OnRamp runs based on the adapter ligation method: transposase based or restriction digest based (Fig. 1A). The first uses ONT's rapid sequencing kit, which uses a transposase to randomly fragment equimolar pooled plasmid DNA and simultaneously ligate ONT's specialized sequencing adapters, to provide compatibility with typical nanopore protocols. In the second, plasmids are linearized with a single-cutter restriction enzyme (RE), which allows for control of both the number and locations of cuts within the plasmid, increasing the likelihood of obtaining full-length plasmid reads compared with the transposase-based approach, and which is used for preparation of plasmid pools containing clonal or highly similar plasmid copies. Restriction-digested plasmids are pooled in equimolar amounts after digestion, end-repaired, and monoadenylated, and then, adapters are added to plasmid ends using ONT's ligation kit.

**Figure 2.**
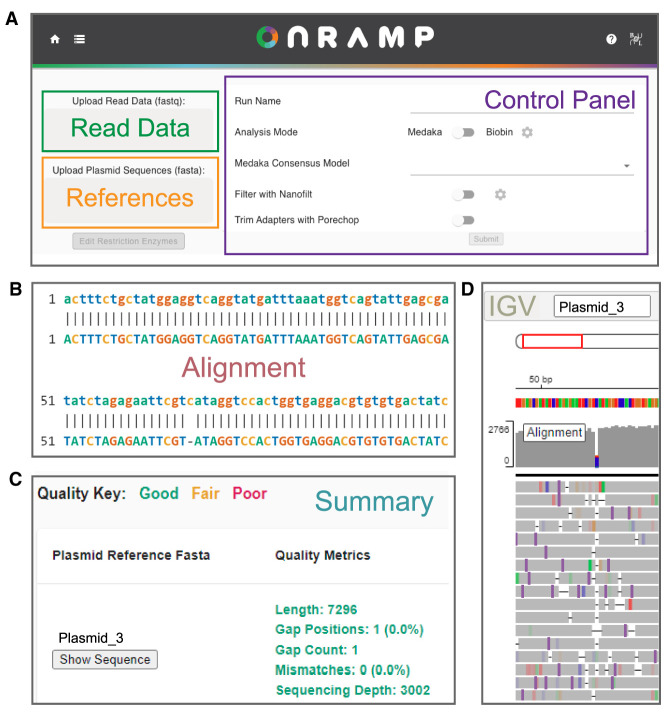
OnRamp web app. (*A*) Image of OnRamp submission page, where users submit read data and plasmid reference files and choose analysis settings. (*B*–*D*) Output generated from example data, including sequence alignments (*B*), alignment quality metrics (*C*), and IGV viewer panel showing individual reads (*D*).

Following adapter ligation, plasmid libraries are loaded onto primed Flongle flow cells and deeply sequenced to base-pair resolution over 16–24 h using ONT's MinION sequencing platform with a Flongle adaptor (Fig. 1B) with single reads spanning entire plasmids. Base-called read files generated by a nanopore run are then submitted to the OnRamp web app along with plasmid reference sequence files for analysis. The pipeline run by the web app aligns reads using medaka (https://github.com/nanoporetech/medaka) to generate a consensus sequence for each plasmid by aligning reads to user-provided references (Fig. 1C), and then consensuses are aligned to their matched references using EMBOSS Needle (Rice et al. 2000) to generate optimal global pairwise alignments (Fig. 1D). After submitting a run on the OnRamp web app (Fig 2A), users are given outputs that include a sequence-level alignment between reference and consensus files (showing any insertions, deletions, or base substitutions) (Fig. 2B); a quality score based on number and length of insertions or deletions (gaps) or base substitutions in the consensus relative to the reference (Fig. 2C); and an Integrative Genomics Viewer (IGV) (Thorvaldsdóttir et al. 2013) view showing read alignments used to generate the consensus (Fig. 2D).

### OnRamp detects base-pair level variation in simulated data sets

To assess the ability of our OnRamp pipeline to accurately detect sequence variation occurring in plasmids from a mixed plasmid pool, we first constructed simulated read data using NanoSim (Yang et al. 2017), a tool designed to simulate nanopore reads. Read libraries were constructed for 30 dissimilar plasmids (average length, 4.4 kb) of known sequence and simulated to be prepared using the ONT transposase rapid adapter kit, giving randomly distributed read start sites. NanoSim generated 29,984 reads, with an average of 967 reads per plasmid, which were pooled and then mapped back to their respective references using OnRamp in medaka mode. In medaka mode, OnRamp uses the medaka consensus tool to generate polished consensus sequences by simultaneously mapping all reads against all references to generate the best alignments, using reference sequences in place of a draft genome assembly. OnRamp mapped, on average, 614 reads to each plasmid, which were used to create 30 consensus sequences. Across these 30 sequences, a total of three errors (two missing single bases at the start of one consensus owing to lack of depth, and a 1-bp gap at a homopolymer run in another plasmid) were observed upon alignment back to their reference sequences (one error per 10 plasmids). Because no errors were expected given these reads originated from known sequences, we tested what level of read coverage would eliminate these gaps. We repeated consensus construction using 500%, 100%, 50%, or 10% of the 29,984 reads and measured gaps in the resulting alignments (Supplemental Fig. S1A). Alignment accuracy varied with read coverage as expected, with more coverage giving increasing accuracy. Consensus errors consisted primarily of gaps at homopolymers and missing sequence at consensus ends owing to unequal coverage across the alignments (Supplemental Fig. S3). Additionally, increased errors in calling homopolymers is a known limitation of ONT data (Rang et al. 2018).

Next, we used this simulated data set to test OnRamp's ability to detect indels. A simulated read pool generated from a reference plasmid containing a 100-, 10-, or 1-bp insertion or deletion was added to the 30-plasmid read pool. We used OnRamp to generate polished consensuses as above, and results showed that insertions and deletions of 100 bp, 10 bp, and 1 bp were all correctly identified, even at 100 reads per plasmid (Supplemental Fig. S1B–E). Read count did not impact ability to detect mutations but rather affected whether additional variation occurred elsewhere in the consensus (points above the dotted lines in Supplemental Fig. S1D,E) as a result of lack of coverage, especially at map ends and homopolymers.

### OnRamp correctly assigns reads to highly similar plasmids

We next used simulated data to test OnRamp's ability to correctly assign reads originating from a pool of plasmid sequences with high sequence identity without using barcoded sequencing adapters. We created an average of 971 reads for each of the 16 plasmid references differing only by 24-bp-, 1-bp-, or 6-bp-long unique regions and used NanoSim to construct simulated read pools. OnRamp was then used to analyze reads and references using biobin mode. In biobin mode, OnRamp scans all provided reference sequences for unique sequence to use for distinguishing the references and then aligns each read to these unique regions to obtain an alignment score. Two tunable alignment scores, context and fine map, are used to assign each read (see Methods) (Supplemental Fig S2A,B). Each read that meets the scoring criteria is binned to this reference, and then OnRamp generates a consensus for each plasmid individually from its assigned bin of reads using medaka's consensus tool. Using biobin mode, fewer than 6% of reads were assigned to the incorrect reference (Supplemental Fig. S2C), and OnRamp was able to generate consensus sequences for pools containing plasmids that differed only by the 12-bp or 24-bp markers. For the 6-bp marker, consensus sequences contained many more gaps owing to a low number of assigned reads. We also ran this test using OnRamp's medaka mode (Supplemental Fig. S2D); however, this is not recommended as medaka mode uses nonuniquely assigned reads in consensus generation and can lead to read misassignment. We suggest using OnRamp's biobin mode for correctly mapping highly similar plasmid pools in which there is at least 24 bp of unique sequence to differentiate the plasmids. For highly similar plasmid pools with fewer than ∼24-bp unique sequences (e.g., plasmids that are clonal copies), a simple alternative to the plasmid preparation protocol is provided below that works for any amount of similarity.

### Nanopore plasmid sequencing reveals mutations in real plasmid data

**Figure 3.**
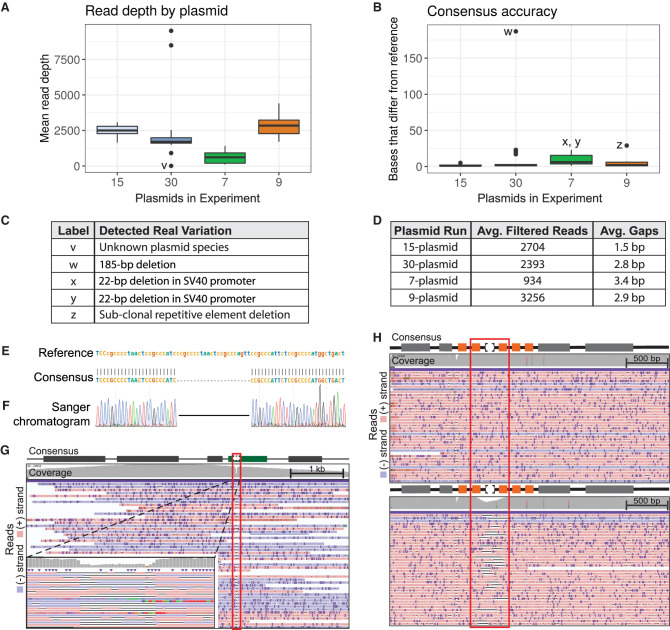
Real plasmid sequencing experiment characteristics and variant detection. (*A*) Per-plasmid read depth across pooled sequencing runs. (*B*) Per-plasmid count of bases in consensus sequence that differ from reference (gaps). (*C*) Table describing variation contributing to outliers (labeled points) in *A* and *B*. (*D*) Table summarizing read and gap data for experiments shown in *A* and *B* (gap counts do not include variants listed in *C*). (*E*) OnRamp alignment results showing a 22-bp deletion. (*F*) Sanger validation of deletion in *E*. (*G*) IGV browser view of reads mapping to deletion (red outline) from *E* in an SV40 promoter (green box). (*Left inset*) Zoomed view. (*H*) IGV view of reads mapping to a clone without (*top*) or a clone with (*bottom*) a subclonal repetitive element (orange boxes) deletion (red outline). IGV: Black lines are deletions; dark purple marks are insertions.

To evaluate the performance of OnRamp with sequencing of real plasmids, we ran four separate plasmid pools containing plasmids of a variety of sequences, similarity levels, and sizes, using both the transposase- and restriction-based protocols; nanopore-sequenced them; and analyzed them using OnRamp's pipeline. A seven-plasmid pool was prepared using the transposase from ONT's Rapid Sequencing Kit. This experiment generated 6539 reads that passed guppy's quality filtering, an average of 934 uniquely assigned reads per plasmid (Fig. 3A) and a consensus accuracy of 3.4 gaps per plasmid on average (Fig. 3B,D), as measured by per-base differences in consensus versus reference.

The high read coverage and read length generated allowed us to distinguish reads and generate consensuses from three highly similar plasmids in this run that differed only by two 4-bp sequences. Additionally, real sequence variation was detected in this run (not included in the per-plasmid gap average). A 22-bp deletion, too small for detection by diagnostic digest and gel electrophoresis, was identified in the SV40 promoter of two plasmids (Fig. 3C,E) and validated by Sanger (Fig. 3E–G). This deletion occurred outside of a region manipulated by molecular cloning and would not normally have been checked and caught by Sanger sequencing.

The nine-, 15-, and 30-plasmid pools were prepared using the restriction digest and ligation method. In the nine-plasmid pool, plasmids had more than 1000 reads per plasmid, with on average 3256 quality-filtered reads per plasmid and an average of 2.9 gaps per plasmid consensus (Fig. 3A–D). As in the simulated data, some of these gaps were from homopolymer errors and were likely related to known issues with correctly calling homopolymers in ONT data (see Discussion). In this run, we were able to sequence through 4× and 6× 40-bp repeats in six of the plasmids that were previously intractable to Sanger sequencing owing to high secondary structure. The high read coverage obtained on these runs allows us to identify subclonal populations in plasmid sequences, which can occur as a result of plasmid recombination during bacterial growth. We detected a subpopulation-level deletion of one of these repeats in a plasmid from this run using OnRamp (Fig. 3C,H). This high coverage is reflected even in the 30-plasmid pool, which averaged 2393 quality-filtered reads per plasmid and minimum of 900 reads per plasmid, generating base-pair resolution for all but one plasmid. This single exception was expected as it was known to have failed a diagnostic restriction digest check, indicating its sequence likely would not match the provided reference. This run had 2.8 gaps per plasmid on average, excluding a 185-bp deletion detected in one plasmid (Fig. 3B,C).

### Validating plasmid sequences in pooled plasmid clones

**Figure 4.**
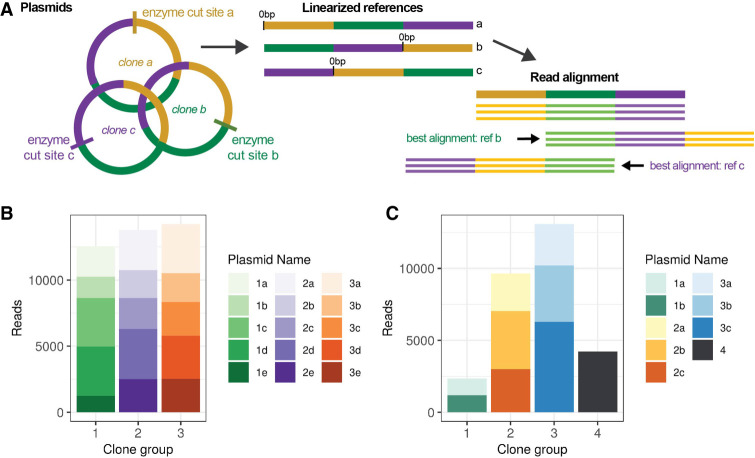
Restriction-digest barcoding for highly similar or clonal plasmids. (*A*) Diagram of restriction cut-site method for unique read mapping of clonal plasmids using “rotated” references. (*B*) Number of reads mapping uniquely to each plasmid in a 15-plasmid clonal test pool. (*C*) Number of reads mapping uniquely to each reference in a nine-plasmid mixed clonal run.

The nine- and 15-plasmid runs described above each contained some plasmids that were clonal copies of each other. Normally, as a result of plasmid pooling, reads originating from different clones of the same plasmid (or highly similar plasmids) would all map to the same reference, making differentiation of the read source impossible without barcoding. However, we were able to successfully leverage nanopore's long read length in a simple alternative restriction-based protocol to differentiate reads originating from identical clones in the same pool without the need for barcoding. For plasmid libraries containing multiple plasmid clones or highly similar plasmids (≤24-bp difference), each clone is cut with a different unique RE from its matched partners before pooling (Fig. 4A). During analysis, a copy of the same plasmid reference sequence is provided for each clone, except with the linear sequence origin set at the digest site used for that clone (termed “rotated” reference). Although each cut clone contains the same total sequence, the alternate digest sites create linear fragments (reads) that map precisely to their matched “cut” reference sequence but poorly to the same sequence reference “cut” at any other site (Fig. 4A). This approach is feasible owing to the long-read nature of nanopore sequencing, in which the majority of reads span an entire plasmid.

We validated this approach experimentally using three ∼6.5-kb plasmids (Fig. 4B, 1, 2, and 3) which are identical except for a ∼500-bp region. Five clones of each of the three plasmids (Fig. 4B, a–e) were digested using different REs for clones within a set (with the closest-cut sites 579 bp apart), pooled, prepared, and sequenced. An average of 2704 reads uniquely mapped when using rotated references (Fig. 4B) compared with seven reads uniquely mapping to nonrotated references, indicating that using different cut locations with clones is sufficient to create reads that align uniquely to their matched rotated reference. The nine-plasmid run contained three sets of clones and one unique plasmid (Fig. 4C). The subpopulation-level deletion of a repetitive element discussed above (Fig. 3G) was detected in a plasmid that was part of a set of three clones in this run.

## Discussion

Assessing recombinant plasmid sequence fidelity is an integral part of any molecular cloning workflow. Although Sanger sequencing is an elegant, cost-effective method for low-throughput plasmid validation, it can be inadequate for multi- and whole-plasmid sequencing and handles regions with complex secondary structure poorly. As an alternative, HTS's run cost, equipment cost, and sample coordination requirements make it inefficient for standard plasmid validation workflows outside of large plasmid libraries. Additionally, HTS requires amplification and, because of its short-read nature, cannot identify and correctly assign mutations outside unique regions in highly similar plasmid pools. With the introduction of ONT's sequencing platforms, sequencing of many plasmids in their entirety at high read depth is now possible. Although some techniques have been published for recombinant plasmid verification using ONT, they rely on transposase barcoded libraries and de novo assembly to validate plasmid libraries (Currin et al. 2019; Emiliani et al. 2022) or are not feasible for sequencing many plasmids simultaneously at comparable costs to Sanger (Brown et al. 2023). Additionally, these approaches all require some degree of programming for use of their analysis pipelines. Tools are needed for analysis of nanopore-based plasmid sequencing data that are accessible to a broad spectrum of researchers, without the need for training in bioinformatics, and that facilitate interpretation of results, in order for full-plasmid nanopore sequencing to become more widespread as an option for validation.

OnRamp combines both wet-laboratory protocols for pooled plasmid preparation using the ONT platform and an associated reference-based computational pipeline packaged in the OnRamp web app, which is designed specifically to support validation of plasmid pools. ONT's compact benchtop sequencing platform is much more affordable than most HTS sequencing platforms and allows for in-laboratory sequencing with results available as soon as the next day, without the need to ship samples to a core or company. OnRamp provides a rapid (0.5–2 h for preparation, 16–36 h for results) and cost-effective approach for medium-throughput plasmid sequencing. Using the reagents and protocols described here, we were able to fully sequence 30 plasmids at $1.25 per kilobase, significantly less than the cost of using Sanger sequencing to obtain equivalent data. Although we did not test larger plasmid pools and used low-pore count Flongles (max, 47 pores), we estimate that these protocols could be used to sequence up to 100 5-kb plasmids at 2000× read depth at a cost of $1.60 per plasmid (or equivalently $0.16/kb for 10 kb plasmids) (for run cost comparisons, see Supplemental Fig. S4). Additionally, the OnRamp web app facilitates analysis and data interpretation in a manner that is accessible to laboratories without the need for extensive bioinformatics support.

Testing OnRamp using simulated read libraries showed its ability to correctly assign sequencing reads to reference sequences and construct consensus sequences even with highly similar plasmids (only 24-bp difference). Testing OnRamp on real plasmid runs showed that OnRamp provides high sequence read depth across plasmid pools, generating consensus sequences spanning entire plasmid lengths at base pair resolution (even on Flongle flow cells with as few as 20 pores). The read depth we obtained (more than 900 reads per plasmid for a 30-plasmid pool) using even ONT's lowest-capacity flow cell (the Flongle) allows for high confidence in base-level calls in consensus sequences despite nanopore's 10% error rate (Chandak et al. 2020). The high coverage we obtained also allowed for detection of subpopulation-level variation even in a region with complex secondary structure and high clonal similarity (Fig. 3H). These mixed populations will not be represented in consensus files; however, they can be detected by viewing the read alignments in IGV, which are provided as part of OnRamp's output. This also allows users to interrogate underlying read data to determine confidence in consensus sequence base and indel calls. This is similar to Sanger sequencing results, in which sequence files do not show subpopulation structure, but trace files might. Additionally, this mutation (a deletion of one of a 6× repetitive element set) likely occurred as a result of bacterial recombination after cloning, underscoring the importance of obtaining full-plasmid sequencing data rather than running spot-check validations using Sanger. These experiments also revealed a 22-bp mutation in a functional noncoding plasmid element (SV40 promoter) that was previously undetected by diagnostic restriction digests, showcasing the ability of the tool to determine uncharacterized structural and sequence variation.

A limitation of Sanger sequencing is the tendency for errors to occur after homopolymer sequences (sequences with repeats of the same base) (Kieleczawa 2006). Although this type of error was also detected in our simulated and real plasmid data, consistent with reports of these errors in ONT sequencing data (Rang et al. 2018), ONT has worked to address this issue, improving homopolymer sensitivity with their new R10 pore chemistry, which should reduce the rates of errors in homopolymers up to 10 bp in length (Sereika et al. 2022).

Using OnRamp's medaka mode to generate consensus sequences, we were able to rapidly validate our plasmids based on alignments to reference sequences. Some limitations of this approach arise as a result of medaka being a reference-based approach as opposed to an assembly-based method. OnRamp uses a reference-based system for analysis, as it is a tool designed specifically for routine plasmid validation. Although this precludes the use of OnRamp for de novo assembly of unknown plasmid sequences (an uncommon case in routine screening), there already exist well-designed tools for de novo assembly of nanopore data (Currin et al. 2019; Emiliani et al. 2022; Brown et al. 2023). For instance, although we were able to detect most variation in our constructs, consensus sequences for plasmids with very large indels (>1000 bp) or where large portions of the plasmid have inserted backward relative to the reference could not be generated. However, these large rearrangements should be easily detected by complementary diagnostic restriction digest tests, which are often a routine step in cloning protocols. Using the alternate biobin mode, choosing unique regions in the reference is essential to binning reads. Indels in the unique portion of the reference can lead to incorrectly binned reads or failure to generate a consensus. An alternative method is to use the clonal restriction-based method we described to separate reads from highly similar or even identical plasmids.

Although we designed OnRamp specifically to make reference-based full-plasmid sequence validation rapid, affordable, and widely accessible to a variety of laboratories in order to facilitate standardization of routine full-plasmid validation, we can imagine a number of other potential applications of this technology. Because of nanopore's ability to sequence through repetitive regions, we can improve our understanding of the rates and sequence dependencies of bacterial recombination-based errors in repetitive sequences during plasmid production and advance our understanding of the behavior and stability of genomic repetitive sequences modeled in plasmids. Additionally, studies might be made possible to improve the reproducibility of plasmid-based research on regulatory element activity by characterizing previously undetected variation in plasmid backbones both within and across studies.

In summary, OnRamp offers rapid, medium-throughput full-plasmid sequencing without secondary structure limitations or the need for primers. It provides more affordability and simplicity than HTS and, with our streamlined web application, makes analysis and interpretation of results accessible and straightforward.

## Methods

### Vector construction and maintenance

Plasmids were constructed using either EMMA (Martella et al. 2017) or gateway- or restriction-based cloning methods. The EMMA toolkit was a gift from Yizhi Cai (Addgene kit 1000000119). Various parts from the toolkit were used for construction of the vectors, and mCherry was cloned from pHR-SFFV-KRAB-dCas9-P2A-mCherry to become a usable part. pHR-SFFV-KRAB-dCas9-P2A-mCherry was a gift from Jonathan Weissman (Addgene plasmid 60954; RRID:Addgene_60954) (Gilbert et al. 2014; http://n2t.net/addgene:60954). Expression vectors were grown in either Stbl3 or DH5ɑ chemically competent *Escherichia coli* strains.

### Transposase-based plasmid preparation

For transposase-based preparation, plasmids were treated using the rapid sequencing kit and following ONT's protocol (SQK-RAD004). Pooled plasmid DNA is brought to 7.5 µL using H_2_O, combined with 2.5 µL FRA, incubated 30°C for 1 min and then at 80°C for 1 min, and then put on ice. One microliter of RAP is added and mixed by flicking, spun down, and incubated for 5 min at room temperature. DNA is loaded onto a primed flow cell.

### Plasmid pool linearization by RE and end-repair

Plasmid DNA was isolated using the QIAprep spin miniprep kit following the manufacturer's protocol (Qiagen 27104) and eluted in water. Plasmids were linearized by restriction digest using a unique cut site, with times, temperatures, and reaction volumes varied for other enzymes according to NEB recommendations. An example pooled restriction digest, NEB Buffer 3.1 (NEB B7203S), was added to 1×, and the final volume was adjusted with nuclease-free water to 200 µL. SwaI (NEB R0604L) was added according to the total amount of DNA present for linearization (minimum, 10 units enzyme per 1 µg DNA), and the sample was digested for 30 min at 25°C. Plasmid pools were generated before digest if all contained the same unique restriction site or were generated after digest for plasmid pools where each plasmid required a different RE. For plasmids in which different REs are used on each plasmid, heat inactivation of each enzyme (following the manufacturer's instructions) or, if not possible, column cleanup (QIAquick PCR purification kit, Qiagen 28104) to remove enzyme was performed and is a crucial step before pooling to prevent cross-cutting of other plasmids in the pool after combination by still-active enzymes.

Digested plasmids were diluted and pooled into a single 1.5-mL microcentrifuge tube using the following rules to calculate desired amount of each plasmid: (1) using an equimolar amount of each plasmid, (2) using a maximum of 1000 ng total plasmid for the entire pool, (3) using at least 10 ng of each plasmid, and (4) using a total 50 µL volume. The amount of each plasmid in a pool ranged from 15–100 ng across experiments in this paper. If any digests generated 3′ or 5′ overhanging bases, pooled plasmids were end-repaired using 1 µL (5 U) DNA Polymerase I Klenow fragment (NEB M0210S) with 33 μM each dNTP and 1× NEB CutSmart buffer per 1000 ng DNA pool, with incubation for 15 min at 25°C and heat inactivation for 20 min at 75°C. Following digestion and end repair, A-tailing was completed using 1 µL of 10 mM dATP and Taq DNA polymerase (NEB M0273S) per 50 µL of sample with incubation for 15 min at 75°C.

### ONT adaptor ligation

For restriction-prepared enzymes, following DNA linearization, end-repair, and A-tailing, ONT's ligation sequencing kit was used (ONT SQK-LSK109) to add adaptors. One half volume of ligation buffer (4× T4 ligase buffer, 60% PEG 8000), 5 µL of T4 DNA ligase (NEB M0202M), and 2.5 µL of AMX (ONT SQK-LSK109) was added to the plasmid mixture and then incubated on a tube rotator for 10 min at room temperature. One volume of 1× Tris-EDTA buffer (pH 7.5; Invitrogen 15567027) and 0.3× room temperature SPRI beads (Beckman Coulter B23317) were added for selection of >2-kb fragments. The sample-SPRI bead mix was incubated on a tube rotator for 10 min on the bench at room temperature. The SPRI beads were washed twice with 100 µL of long fragment buffer (LFB; ONT SQK-LSK109), and the sample was eluted in 9 µL of elution buffer (EB; ONT SQK-LSK109).

### Nanopore sequencing

Flongle flow cells were loaded into minION sequencers containing Flongle adaptors from ONT. Flow cells were primed for the sequencing runs following ONT's standard protocol, using flow cell priming buffers provided by ONT. Briefly, flow cells are quality-checked to check for a usable number of active pores (∼0.5 to one pore per plasmid was used here as the minimum). Flow cell was washed with FB and then SQB buffer mixed 1:1 with water. DNA prepared from previous steps is mixed with SQB and LB immediately before loading following ONT's protocols.

### Simulated reads

NanoSim was used to construct pooled plasmid read libraries. First, a model was created using 81,070 reads (N50 = 6003 bp) from a previous plasmid sequencing experiment, and the 30 plasmid sequences (average length = 4318.7 bp) were used as the reference genome and input in the characterization set. This model was then used to simulate reads from other plasmid references and from references constructed with 1-, 10-, 100-, and 1000-bp deletions and insertions of random sequence, as well as plasmids with 6-, 12-, and 24-bp unique regions.

### Bioinformatics pipeline

Base-calling was completed using Guppy (ONT, 4.5.2) using the dna_r9.4.1_450bps_hac.cfg configuration, and passing reads (Q≥ 7) were filtered using Guppy or NanoFilt (De Coster et al. 2018). Adapters were trimmed using Porechop (https://github.com/rrwick/Porechop). The OnRamp web app allows users to use Porechop and NanoFilt to trim reads and filter by their chosen *q*-score and read length. Reference sequences were generated using SnapGene (https://www.snapgene.com/). The reads and references were then used as input for OnRamp during pipeline testing and development. The OnRamp pipeline and web tool are then run in either medaka, or binning mode, as detailed below.

The medaka mode uses ONT's medaka (https://github.com/nanoporetech/medaka, version 1.4.4) to create consensus sequences and should be used for mixed plasmid pools or for clonal pools prepared by RE digest (detailed in section Validating Plasmid Sequences in Pooled Plasmid Clones). The medaka consensus module was used to generate consensus sequences from read pileups using the “-g” flag to stop filling in gaps with draft/reference sequence during consensus stitching.

The binning mode is used for very highly similar sequences, such as those with a small unique identifier. The biobin module mode of plasmid sequencing was used to bin reads based on unique sequences in the provided references. The biobin mode/module searches the reference sequences for unique sequences >3 bp, and a set is constructed for each plasmid reference. Each input read was then aligned to these regions using Biopython pairwise aligner with the following alignment parameters: match, 3; mismatch, -6; open_gap, -10; extend, -5. Reads were first aligned to an extended portion of the plasmid containing 20 bp flanking the unique region and assessed using the “context score.” For reads that passed this threshold, the aligned portion was then aligned and scored against the exact unique region, and high scoring reads (fine score > 80) were assigned to the plasmids. Each of the resulting bins was then passed to medaka for consensus polishing.

The resulting alignments are then filtered (MAPQ ≥ 10) for visualization using the IGV (Thorvaldsdóttir et al. 2013). Final pairwise alignments were constructed between the reference and consensus sequences generated by medaka using EMBOSS needle (EMBOSS:6.6.0.0).

## Data access

The OnRamp is available through a web app at https://onramp.boylelab.org/. The command line version and pipeline used for the application are available at GitHub (https://github.com/Boyle-Lab/bulk_plasmid_seq_web and https://github.com/crmumm/bulkPlasmidSeq). OnRamp source code is also available as Supplemental Code. All plasmid read data and references generated in this study are available at Zenodo (https://doi.org/10.5281/zenodo.7595170). FASTA files are also available as Supplemental Material.

## Supplementary Material

Supplemental Material
