# Supplementary material for: Multiplexed long-read plasmid validation and analysis using OnRamp
Source: Genome Res. 2023 May;33(5):741–9. doi: 10.1101/gr.277369.122 (PMC10317119; doi:10.1101/gr.277369.122)
Supplement: Supplemental Material [file supp_33_5_741__DC1.html]

Multiplexed long-read plasmid validation and analysis using OnRamp — Supplemental Material 

# Multiplexed long-read plasmid validation and analysis using OnRamp

## Supplemental Material

- Supplemental\_Material\_FINAL.pdf
- SUPPLEMENT\_OnRamp\_1.0.0.zip
- SUPPLEMENTAL\_plasmids\_ref\_7.fasta.zip
- SUPPLEMENTAL\_plasmids\_ref\_9.fasta.zip
- SUPPLEMENTAL\_plasmids\_ref\_15.fasta.zip
- SUPPLEMENTAL\_plasmids\_ref\_30.fasta.zip
