# Supplementary material for: Multiplexed long-read plasmid validation and analysis using OnRamp
Source: Genome Res. 2023 May;33(5):741–9. doi: 10.1101/gr.277369.122 (PMC10317119; doi:10.1101/gr.277369.122)
Supplement: Supplemental Material [file supp_gr.277369.122_Supplemental_Material_FINAL.pdf]

Supplement for
“Multiplexed long-read plasmid validation and analysis using OnRamp”

Camille Mumm[1,3], Melissa L. Drexel[1,3], Torrin L. McDonald[2], Adam G. Diehl[2], Jessica A. Switzenberg[2], Alan P. Boyle[1,2,✉]

1. Department of Human Genetics, University of Michigan, Ann Arbor, MI, USA 48109
2. Department of Computational Medicine and Bioinformatics, University of Michigan, Ann Arbor, MI, USA 48109
3. These authors contributed equally to this work.
✉ **Correspondence: apboyle@umich.edu**

Table of Contents for Supplement

Supplemental Figure S1 …………………………………………….. p2

Supplemental Figure S2 …………………………………………….. p3

Supplemental Figure S3 …………………………………………….. p4

Supplemental Figure S4 …………………………………………….. p5

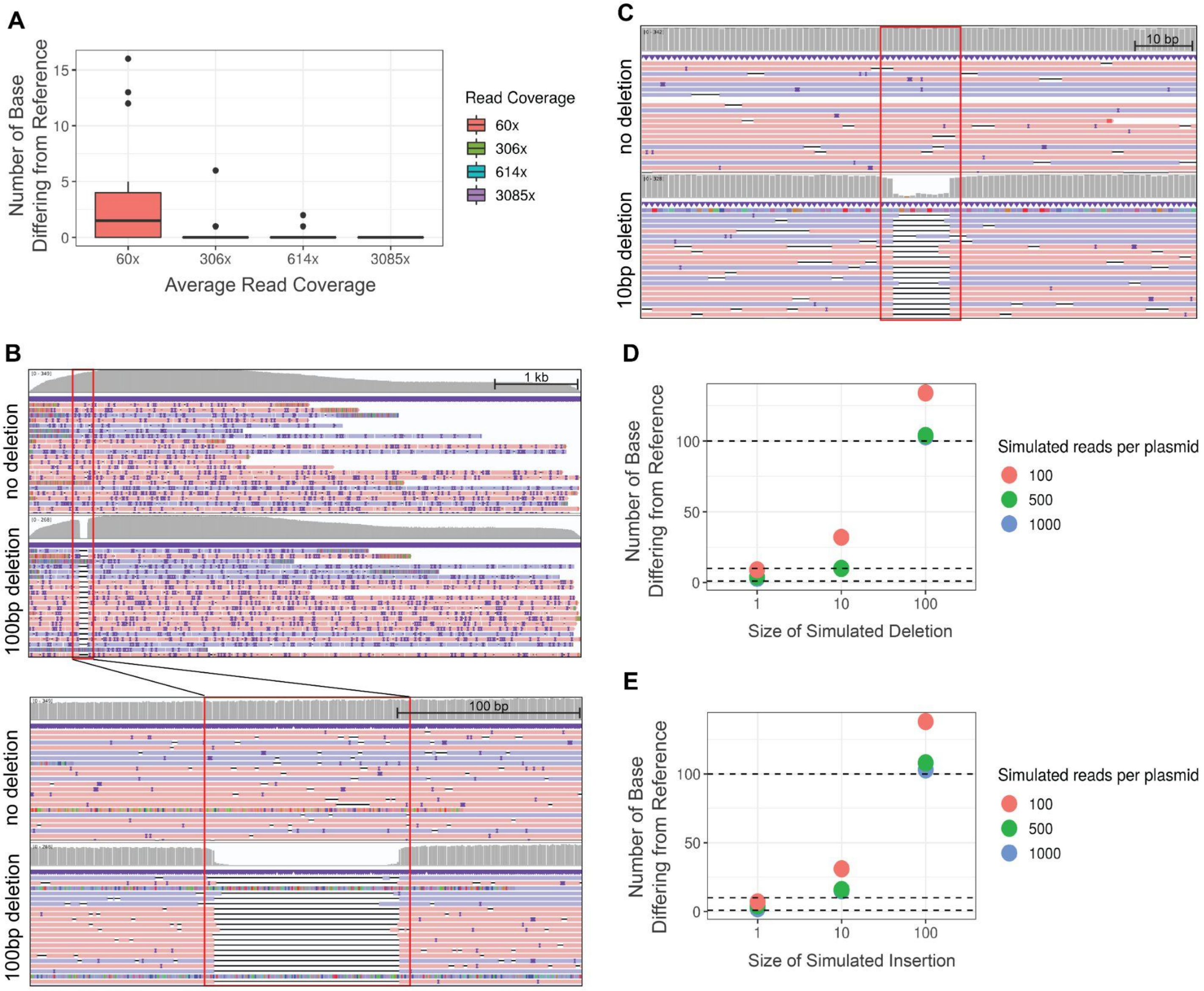

Supplemental Figure S1. Detecting insertions and deletions in a plasmid pool using a simulated read library

a. Consensus accuracy vs read coverage - number of gaps in consensus sequence (before indels) for simulated read depth experiments at various read coverage. b and c. IGV view of read pileups for reads with vs reads without a 100 bp deletion (b) and a 10 bp deletion (c). Deletions highlighted by red boxes. Gray top row shows read depth at each position. Below, minus-strand reads (purple) and plus-strand reads (red) with inserts (dark purple) and deletions (black). d and e. Number of base-pair differences between reference and consensus files for each simulation condition at different read depths. Dotted lines indicate expected number of differences due to simulated deletion (d) or insertion (e).

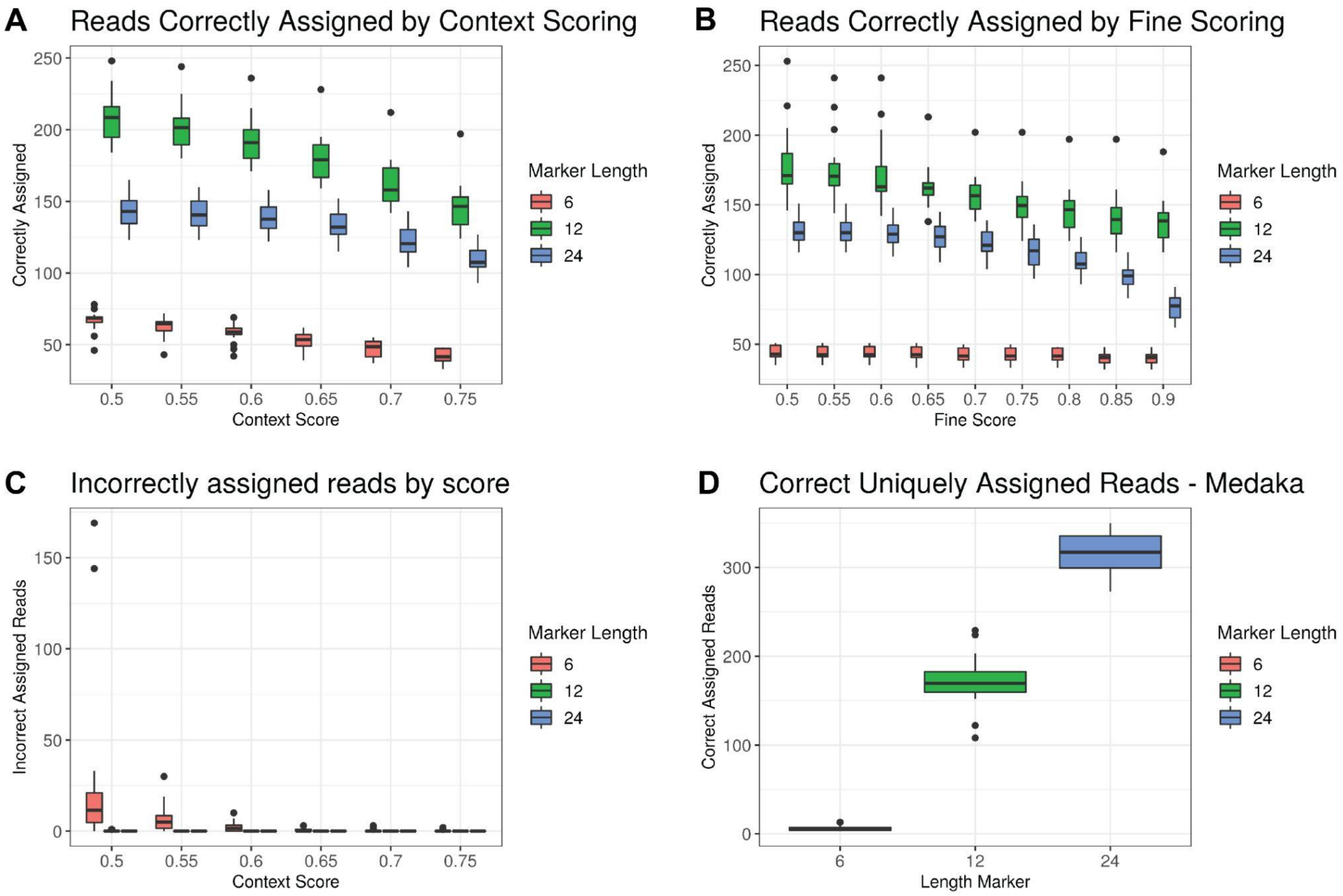

Supplemental Figure S2. Number of correctly assigned reads for each of the 30 simulated plasmids containing a 6bp, 12bp, or 24bp unique region using different modes

Reads aligned using biobin mode (a,b) with a fixed fine score, comparing read counts at different context scores (a) or with a fixed context score, comparing read counts at different fine scores (b). The number of reads incorrectly assigned using different context scores (c). Count of uniquely mapping, correctly assigned reads using medaka mode (d).

A Transposase-prepared plasmid

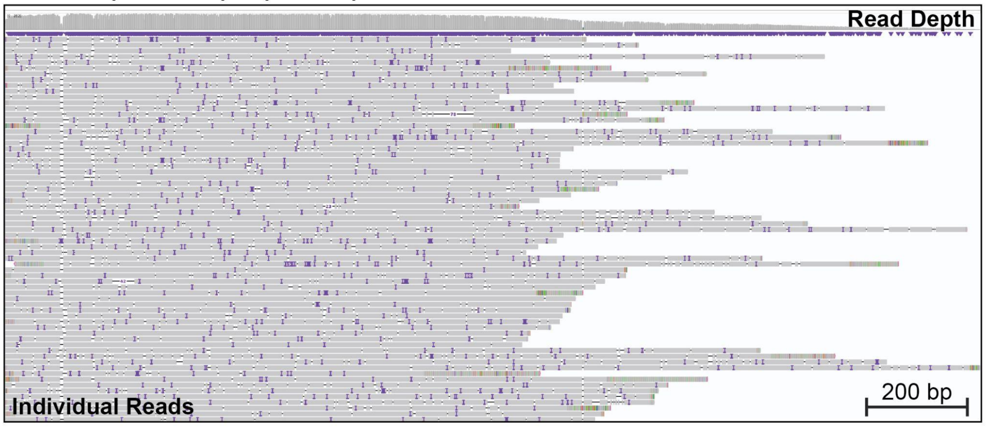

B Restriction-digested plasmid

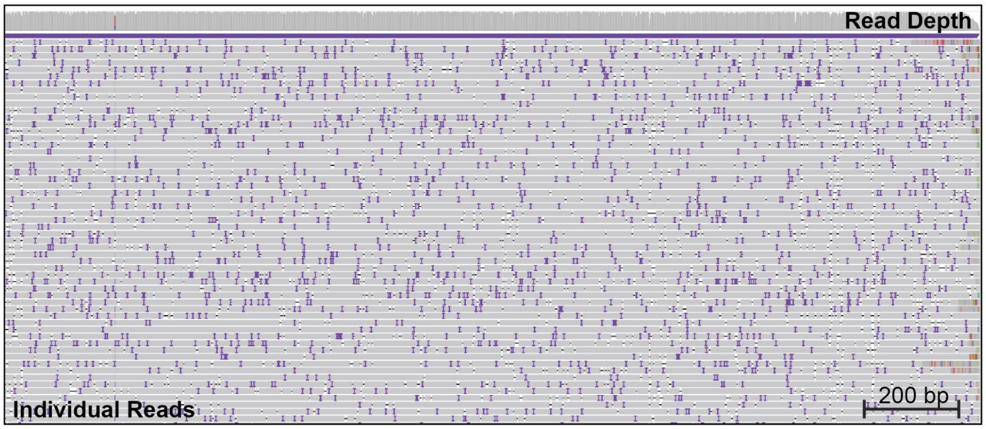

Supplemental Figure S3. Difference in read coverage at plasmid ends with transposase vs restriction digest preparation

IGV view showing read coverage (uniquely mapped reads) for the end of a plasmid simulated to be prepared using transposase (a) or restriction digest (b) with read coverage indicated by height of gray panel at top.

	Plasmids	Sum reference lengths (kb)	Pores	Yield (Mb)	Cost per reference kb	Equivalent cost per kb for Sanger*
Runs in this study	7	29.5	23	51.6	$4.74	$3.98
	9	38.2	19	165.0	$4.13	$4.51
	15	98.7	34	372.9	$1.60	$3.26
	30	129.6	47	481.7	$1.22	$3.33
	. . .	. . .	. . .	. . .	. . .	. . .
Hypothetical typical Flongle output	100	500	90	1000	$0.32	$3.00
Theoretical maximum Flongle output	. . .	. . .	128	2800	. . .	. . .

**Assuming $3 per kb Sanger cost, one $3.60, 20bp primer designed per kb, and that similar plasmids re-use the same primer set*

Supplemental Figure S4. Comparison of costs for plasmid runs using nanopore vs Sanger
Table showing per-kb cost of plasmid runs from the paper. Values in green represent the cheaper of the two costs. Assumptions made for estimating Sanger costs listed below the table. Nanopore costs were estimated using advertised prices for reagents at the time of experiment, Sanger costs estimated using primer and Sanger costs for the services used by our lab. *We generally used Flongles with less than 47 pores and achieved sufficient coverage. Given a more typical 90-pore Flongle, which could produce 1Gb of sequence data (nanopore estimates a theoretical 2.8Gb max for Flongles), we estimated costs for a hypothetical run with one hundred, 5000bp plasmids at 2000× coverage (1000 was sufficient for our purposes), and estimates are listed in the table for this run in red.*
