# Supplementary material for: Multiplexed long-read plasmid validation and analysis using OnRamp
Source: Genome Res. 2023 May;33(5):741–9. doi: 10.1101/gr.277369.122 (PMC10317119; doi:10.1101/gr.277369.122)
Supplement: Supplemental Material [file supp_gr.277369.122_SUPPLEMENT_OnRamp_1.0.0.zip › On-Ramp_web.1.1.0.zip!/bulk_plasmid_seq_web-1.1.0/client/public/index.html]

On-Ramp


You need to enable JavaScript to run this app.
